# Kinetic Model of Incipient Hydride Formation in Zr Clad under Dynamic Oxide Growth Conditions

**DOI:** 10.3390/ma13051088

**Published:** 2020-02-29

**Authors:** Qianran Yu, Michael Reyes, Nachiket Shah, Jaime Marian

**Affiliations:** 1 Department of Mechanical and Aerospace Engineering, University of California Los Angeles, Los Angeles, CA 90095, USA; yuqianran0709@gmail.com (Q.Y.); mpreyes1974@yahoo.com (M.R.); 2 Department of Materials Science and Engineering, University of California Los Angeles, Los Angeles, CA 90095, USA; nachiketshah@outlook.com

**Keywords:** zirconium corrosion, zircalloy clad, Zr hydride, hydrogen, nuclear reactor

## Abstract

The formation of elongated zirconium hydride platelets during corrosion of nuclear fuel clad is linked to its premature failure due to embrittlement and delayed hydride cracking. Despite their importance, however, most existing models of hydride nucleation and growth in Zr alloys are phenomenological and lack sufficient physical detail to become predictive under the variety of conditions found in nuclear reactors during operation. Moreover, most models ignore the dynamic nature of clad oxidation, which requires that hydrogen transport and precipitation be considered in a scenario where the oxide layer is continuously growing at the expense of the metal substrate. In this paper, we perform simulations of hydride formation in Zr clads with a moving oxide/metal boundary using a stochastic kinetic diffusion/reaction model parameterized with state-of-the-art defect and solute energetics. Our model uses the solutions of the hydrogen diffusion problem across an increasingly-coarse oxide layer to define boundary conditions for the kinetic simulations of hydrogen penetration, precipitation, and dissolution in the metal clad. Our method captures the spatial dependence of the problem by discretizing all spatial derivatives using a stochastic finite difference scheme. Our results include hydride number densities and size distributions along the radial coordinate of the clad for the first 1.6 h of evolution, providing a quantitative picture of hydride incipient nucleation and growth under clad service conditions.

## 1. Introduction

Corrosion of metallic structural materials is a pervasive phenomenon in industry and technology [1,2,3,4]. In nuclear reactors, understanding the kinetics of corrosion of metallic components is grand materials science challenge due to the synergistic combination of high temperature, mechanical stresses, complex coolant and fuel chemistry, and irradiation [5,6,7]. In light-water nuclear reactors (LWR) zirconium alloys are used as cladding material in fuel elements to provide mechanical integrity between the coolant (water) and the fuel while keeping low levels of neutron absorption [8,9,10,11]. In principle, Zr clad is subjected to corrosion from the coolant (water) and fuel sides, both by way of oxygen and hydrogen penetration. The oxidation and hydrogenation of zirconium fuel components in LWR may affect reactor safety and efficiency, which makes corrosion a critical design aspect of Zr materials response in nuclear environments [12,13,14,15,16,17,18].

While the majority of the focus of corrosion studies has centered on oxidation and oxygen transport and chemistry in the clad, in the corresponding temperature range, zirconium is known to absorb hydrogen and form hydrides once the critical concentration is reached in the interior of the clad. The accumulation of hydrides during operation plays an important role in fuel performance and safety during steady-state operation and transients, accident conditions, and temporary and permanent fuel storage [19,20]. Examination of the Zr-H phase diagram below 810 K [21,22,23,24] indicates that the first stable compound that appears after the metal solid solution (α-Zr) is a cubic phase with a nominal stoichiometry of 1:1.5 (atomic) known as δ-hydride. Generally, a range of stoichiometries between 1.52 and 1.66 is accepted experimentally as corresponding to this phase [25,26]. While the presence of other metastable hydrides has been reported depending on temperature, aging time, or alloy composition [27], it is now well accepted that the needle-shaped structures that form in the metal region beneath the oxide layer are δ-hydride precipitates. Precipitation first starts when the hydrogen concentration reaches the terminal solubility limit, which ranges from zero at 523 K to approximately 7% at. at 810 K [21]. Although δ Zr${}_{2}$H${}_{3}$ displays good thermo-mechanical stability, it is also an exceedingly brittle phase [28,29,30,31] that can compromise the clad’s mechanical integrity [18,31,32,33].

A key observation of the hydride microstructure is the elongated shape of the precipitates up to a few microns in length [34,35,36,37], typically aligned along directions consistent with the stress distribution within the clad. Calculations and experiments point to the large misfit strains between the cubic δ-hydride and the host α-Zr as the reason behind such preferential alignment [35,38,39,40,41,42], which may also impact the mechanical response of the clad. Indeed, the formation of brittle hydride phases is a principal cause of delayed hydride cracking (a subcritical crack growth mechanism facilitated by precipitation of hydride platelets at the crack tips in Zr clad [19,43,44,45]).

The phenomenology of corrosion is such that oxidation and hydrogenation are typically treated separately, despite some evidence suggesting that there might exist synergisms between oxygen and hydrogen pickup and transport that must be considered jointly in corrosion of Zr [46,47,48,49,50]. This is partially due to the formation of a clearly distinguishable outer oxide scale and and inner region where hydride platelets accumulate. In keeping with this distinction, existing models of hydrogen pickup and precipitation have been developed assuming no cooperative effects from oxygen on hydrogen transport and reaction [35,51]. Models based on hydrogen supersaturation of the α-Zr metal [52,53,54] assume a binary partition of hydrogen in the clad, either as solid solution or as part of precipitates without specification of their size, number, or orientation. Detailed cluster dynamics (CD) modeling offers a more accurate alternative to obtain hydride size distributions and number densities by solving the complete set of differential balance equations with one-dimensional spatial resolution [55,56]. Phase field methods can capture extra detail by furnishing the shape and orientation of hydrides in addition to concentrations and sizes [27,57,58].

An important aspect often overlooked in the models when studying hydrogen transport and hydride formation in the clad is that it occurs in a dynamic setting, with the oxide scale growing in time and hydrogen traversing an increasingly thicker layer before it can reach the interface. This is rationalized in terms of sluggish H diffusion through the oxide in the relevant temperature range (<300 ${}^{\circ }$C), suggesting that this then would be the rate limiting step [34,36]. This is the accepted picture during the *pre-transition* regime, as, after that, fast H transport then occurs through percolated crack networks formed in the oxide layer [59]. However, there is contradicting evidence in the literature about this [60,61], and it is not clear what effect a dynamic boundary condition might have on hydrogen precipitation in the metal substrate at higher temperatures, and what the evolution of the hydride microstructure will be in those conditions. With the objective of shedding new light on these and other issues by using new computational and experimental understanding, in this paper, we present an comprehensive hydrogen transport and precipitation model in Zr formulated from first principles reaction kinetics and fundamental thermodynamics and mechanics. The model is parameterized using electronic structure calculations and experiments and captures both transport across the oxide layer growth and precipitation in the clad under dynamic hydrogen concentration profiles at the oxide/metal interface. First, we describe the fundamental chemistry and phenomenology of the hydrogen evolution in the clad followed by a mathematical formulation of the model. We then provide numerical results under a number of conditions relevant to LWR operation. We finalize with a discussion of the results and the implications of our modeling approach for zircalloy behavior.

## 2. Chemical Reaction Kinetics Model

### 2.1. Zr-Clad Hydrogen Chemistry

The formation of hydrides in the clad is predicated on exposure of its outer surface to bi-molecular hydrogen. This can occur as a consequence of exposure to water or steam, from the reduction of water molecules as:

(1) $$2HO_{2}+4e^{-}\to 2O^{2-}+2H_{2}$$

or directly from exposure to hydrogen gas. It is well known that only a fraction of the hydrogen produced in this way is absorbed by the clad, ranging between 5 and 20% of the total hydrogen uptake (the total amount of hydrogen obtained stoichiometrically from reaction (1) [35,50,62,63,64]. This, known as the *pickup* fraction, sets the boundary condition for the adsorption of hydrogen at the clad’s surface. Adsorbed H${}_{2}$ molecules can split into atomic hydrogen by a number of processes [65,66], although whether this atomic H appears in a neutral or charged state in the metal is still an issue under debate [61,65]. Hydrogen atoms diffuse through the oxide layer and reach the oxide/metal interface, from which they can enter the α-Zr substrate and undergo a number of processes depending on temperature and concentration. Above the terminal solubility limit, hydrogen and zirconium react to form a hydride:

(2) $$\mathrm{Zr}+xH\to {\mathrm{ZrH}}_{x}$$

where *x* is the atomic hydrogen concentration.

**Figure 1:**
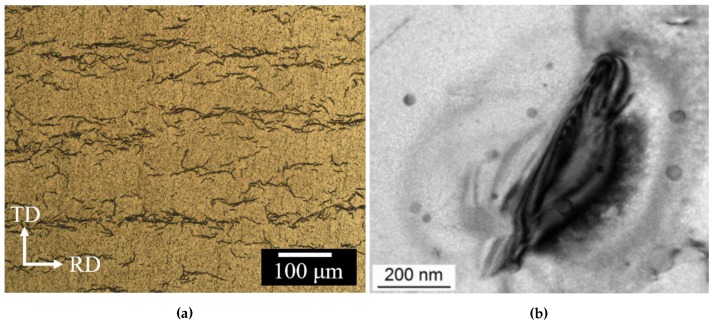
(**a**) Optical micrographs of hydride morphologies in Zircaloy-4. ‘TD’ and ‘RD’ indicate the tangential and radial directions in the clad (from Ref. [67], reproduced with permission of the International Union of Crystallography). (**b**) Electron micrograph detail of a needle-like Zr hydride (reproduced with permission from Ref. [68]).

By way of illustration, Figure 1 shows representative hydridized microstructures in Zirc-4 and Zirconioum.

**Figure 2:**
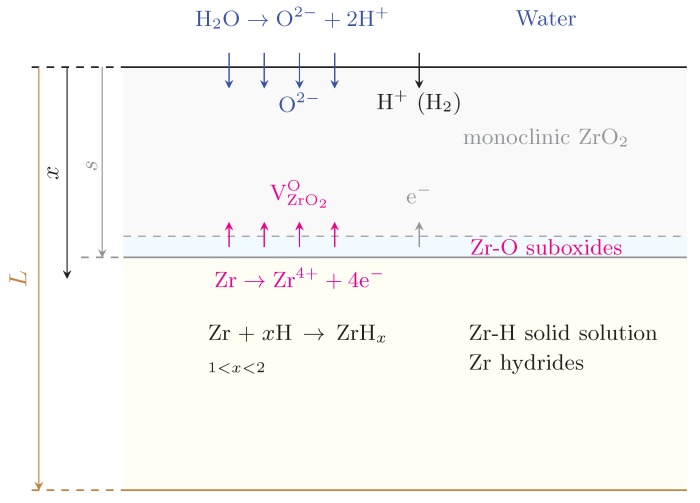
Schematic diagram (not to scale) of the geometry considered for the hydrogen penetration and hydride model developed in this work. *x* is the depth variable, *s* is the thickness of the oxide scale, and *L* is the total thickness of the clad. The chemical processes occurring at each interface are shown for reference.

In view of this picture, and to be consistent with our recent work on oxide layer growth modeling [69], we split our model into two connected elements: (i) a transport part involving H diffusion through an evolving oxide layer, and (ii) a kinetic model of hydride formation and growth in the metal with a dynamic boundary condition set by the first part (i). Figure 2 shows a schematic diagram of the geometry considered for this study and the principal chemical processes taking place in the material. Although it is well known that the Zr oxide layer is not monolithic, containing various Zr-O phases depending on the external conditions and alloy composition [69,70,71,72], here we consider a single phase (monoclinic) ZrO${}_{2}$ with thickness defined by the variable s(t). Hydrogen’s diffusion through this layer is thought to occur mostly along grain boundaries, in microstructures ranging from columnar in out-of-pile [71] tests to roughly equiaxed for in-pile conditions [70]. This phenomenon takes place during the pre-transition regime, before the oxide layer cracks and/or develops porosity due to Pilling-Bedworth stresses developed during the metal-to-oxide transformation [73,74]. Once cracking occurs, new diffusion avenues open up for hydrogen to reach the interface and diffusion is no longer seen as a rate limiting step. Our model applies only up to this transition point but not beyond.

### 2.2. Diffusion Model of Hydrogen in ZrO_2_

The goal of this part of the model is to determine the hydrogen concentration at the metal/oxide interface as a function of time. For this, a generalized drift-diffusion equation is solved:

(3) $$\frac{\partial c_{H}}{\partial t}=\nabla \left(D_{H}\nabla c_{H}\right)-\frac{U_{H}D_{H}}{kT^{2}}\nabla c_{i}\nabla T+\frac{qD_{H}}{kT}\nabla \left(c_{H}\nabla \phi \right)$$

This equation includes the following contributions:

- The first term is standard *Fickian* diffusion in the presence of a concentration gradient.
- The second term is the so-called *thermo-migration* contribution, which depends on the temperature gradient and where $U_{H}$ is the activation energy for diffusion. The convention is for interstitial solutes to move in the direction opposing the gradient, i.e., a ‘negative’ drift contribution in the equation.
- The third term represents *electro-migration*, where *q* is the charge of the diffusing species (+1 for protons), and ϕ is the electrical potential, which can be determined by solving Poisson’s equation:
  (4) $$\nabla^{2}\phi =-\frac{\rho }{\varepsilon }$$
  where ρ is the charge density and ε is the dielectric permittivity.

Consistent with our previous work [69] and other studies [61], we assume the existence of a charge gradient across the oxide layer that originates from the onset of an electron density profile [75]. As well, in this work we consider autoclave conditions and thus neglect the thermomigration contribution.

Equation (3) is solved in one dimension (x) using the finite difference model with the following dynamic boundary conditions:

$$\begin{array}{ccc} c_{H}\left(x,0\right) & = & 0\\ J_{H}\left(0,t\right) & = & D_{H}\frac{\partial c_{H}\left(0,t\right)}{\partial x}=2f_{H}C_{0}\frac{\partial s}{\partial t} \end{array}$$

where $C_{0}$ is the amount of oxygen (per unit volume) absorbed into the clad to form Zr oxide.The first condition trivially states that the hydrogen content in the clad at the beginning of time is equal to zero, while the second one prescribes the flux of hydrogen at the water/oxide interface. This condition is time-varying as indicated by the growth rate of the oxide layer, $\dot{s}$. As well, it depends on the H pickup fraction, $f_{H}$, which albeit may also be time dependent [62,64], we fix at 15% for the remainder of this work. The factor of ‘2’ represents the fact that there are two atoms of hydrogen per oxygen atom available to penetrate the clad. Under homogeneous oxide formation conditions, $C_{0}\approx 2\rho_{\mathrm{Zr}}$, with $\rho_{\mathrm{Zr}}$ the Zr atomic density.

Expressions for s(t) have been provided in our previous study for a number of nuclear-grade Zr alloys [69]. In general, $s\left(t\right)=at^{n}$ such that the growth rate can be directly expressed as

(5) $$\frac{\partial s}{\partial t}=ant^{n-1}$$

*a* values range between 0.33 (pure Zr) and 0.37 (Zirc-4), while n=0.34 in both cases. These values give *s* in microns when *t* is entered in days. With this, $\dot{s}\approx 0.11t^{-0.66}$ (microns per day).

### 2.3. Stochastic Cluster Dynamics Model with Spatial Resolution

Here we use the stochastic cluster dynamics method (SCD) [76] to perform all simulations. SCD is a stochastic variant of the mean-field rate theory technique, alternative to the standard implementations based on ordinary differential equation (ODE) systems, that eliminates the need to solve exceedingly large sets of ODEs and relies instead on sparse stochastic sampling from the underlying kinetic master equation [76]. Rather than dealing with continuously varying defect concentrations $C_{i}$ in an infinite volume, SCD evolves an integer-valued defect population $N_{i}$ in a finite material volume *V*, thus limiting the number of ‘active’ ODEs at any given moment. Mathematically, SCD recasts the standard ODE system:

(6) $$\frac{dC_{i}}{dt}=g_{i}-\sum_{i}s_{ij}C_{i}+\sum_{i}s_{ji}C_{j}-\sum_{i,j}k_{ij}C_{j}C_{i}+\sum_{j,k}k_{jk}C_{i}C_{k}$$

into stochastic equations of the form:

(7) $$\frac{dN_{i}}{dt}={\tilde{g}}_{i}-\sum_{i}{\tilde{s}}_{ij}N_{i}+\sum_{i}{\tilde{s}}_{ji}N_{j}-\sum_{i,j}{\tilde{k}}_{ij}N_{j}N_{i}+\sum_{j,k}{\tilde{k}}_{jk}N_{i}N_{k}$$

The set $\left\{\tilde{g},\tilde{s},\tilde{k}\right\}$ represents the reaction rates of 0th (insertion), 1st (thermal dissociation, annihilation at sinks), and 2nd (binary reactions) order kinetic processes taking place inside *V*, and is obtained directly from the standard coefficients {g,s,k} as:

$$\tilde{g}\equiv gV,\;\tilde{s}\equiv s,\;\tilde{k}\equiv kV^{-1}$$

The value of *V* chosen must satisfy

$$V^{\frac{1}{3}}>\ell$$

where *ℓ* is the maximum diffusion length $l_{i}$ of any species *i* in the system, defined as:

(8) $$\begin{array}{c} \ell =\max_{i}\left\{l_{i}\right\} \end{array}$$

(9) $$\begin{array}{c} l_{i}=\sqrt{\frac{D_{i}}{R_{i}}} \end{array}$$

Here, $D_{i}$ and $R_{i}$ are the diffusivity and the lifetime of a mobile species within *V*. The above expression is akin to the stability criterion in explicit finite difference models. From Equation (7), $R_{i}=\tilde{s}+\sum_{j}{\tilde{k}}_{ij}N_{j}$. The system of Equation (7) is then solved using the kinetic Monte Carlo (residence-time) algorithm by sampling from the set $\left\{\tilde{g},\tilde{s},\tilde{k}\right\}$ with the correct probability and executing the selected events. Details of the microstructure such as dislocation densities and grain size are captured within SCD in the mean-field sense, i.e., in the form of effective sink strengths for hydrogen atoms. For example, the value of $s_{ij}$ in Equation (6) for dislocation and grain boundary defect sinks is:

(10) $$s_{ij}=s_{d}+s_{g}b=\rho +\frac{6\sqrt{\rho }}{d}$$

where ρ is the dislocation density and *d* is the grain size.

**Figure 3:**
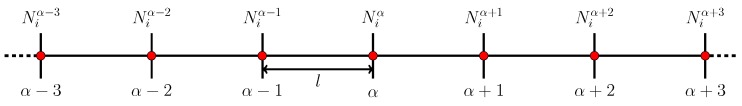
Schematic diagram of two volume elements of a 1D space discretization used to calculate spatial gradients within the stochastic cluster dynamics method (SCD). The superindex α refers to the physical element, while the subindex *i* refers to the cluster species.

SCD has been applied in a variety of scenarios not involving concentration gradients [76,77]. The SCD code has been developed in house and is available at: http://jmarian.bol.ucla.edu/packages/packages.html. However, Equation (6) must be expanded into a transport equation (i.e., a *partial differential equation*, or PDE) by adding a *Fickian term* of the type:

(11) $$\frac{dC_{i}}{dt}=\nabla \left(D_{i}\cdot \nabla C_{i}\right)+f\left(t;C_{1},C_{2},C_{3}\ldots \right)$$

where $D_{i}$ is the diffusivity of species *i*, and $f(t;C_{1},C_{2},C_{3}\ldots )$ is used for simplicity to represent all of the terms in the r.h.s. of Equation (6). To cast Equation (11) into a stochastic form, the transport term must be converted to a reaction rate in the finite volume *V*. As several authors have shown, this can be readily done by applying the divergence theorem and approximating the gradient term in terms of the numbers of species in neighboring elements [78,79]. For a one-dimensional geometry such as that schematically shown in Figure 3, the Fickian term simply reduces to:

$$D_{i}\frac{N_{i}^{\alpha }-N_{i}^{\beta }}{l^{2}}$$

where Greek superindices refer to neighboring elements, *i* is the cluster species, and *l* is the element size. When summed over all neighboring elements, this term then represents the rate of migration of species *i* from volume element α to β, which can be now added to the r.h.s. of Equation (7) and sampled stochastically as any other event using the residence-time algorithm.

The full stochastic PDE system, written for a generic species *i* in volume element α, takes then the following form:

(12) $$\frac{dN_{i}^{\alpha }}{dt}=D_{i}\sum_{\beta }\frac{N_{i}^{\alpha }-N_{i}^{\beta }}{l^{2}}+{\tilde{g}}_{i}-\sum_{i}{\tilde{s}}_{ij}N_{i}^{\alpha }+\sum_{i}{\tilde{s}}_{ji}N_{j}^{\alpha }-\sum_{i,j}{\tilde{k}}_{ij}N_{j}^{\alpha }N_{i}^{\alpha }+\sum_{j,k}{\tilde{k}}_{jk}N_{i}^{\alpha }N_{k}^{\alpha }$$

Further, the model assumes the following:

(i) The only mobile species considered are hydrogen atoms.
(ii) The source term ${\tilde{g}}_{i}$ only applies to element 0 (oxide/metal boundary) and is calculated from the hydrogen arrival flux calculated from the model in Section 2.2.
(iii) The only processes considered in the metal are:
  (a) H diffusion
  (b) Immobilization of H atoms through formation of Zr${}_{2}$H${}_{3}$ molecules (equivalent to nucleation of hydride platelets).
  (c) Growth of Zr${}_{2}$H${}_{3}$ clusters.
  (d) Thermal dissolution of Zr${}_{2}$H${}_{3}$ clusters.

Next, we provide suitable expressions for each of the kinetic processes just listed.

#### 2.3.1. H Atom Diffusion

The hydrogen diffusivity in both the oxide and the metal is assumed to follow an Arrhenius temperature dependence:

(13) $$D_{H}^{\alpha }\left(T\right)=D_{0}^{\alpha }\exp\{\left(-\frac{e_{m}^{\alpha }}{kT}\right)\}$$

where $D_{0}$ is the exponential pre-factor, $e_{m}$ is the migration energy, *k* is Boltzmann’s constant, and the superscript α can refer to the oxide (‘ox’) or the metal (‘m’). The diffusivity of H in Zircaloy-4 oxides has been recently measured by Tupin et al. [80], which give values of $2.5\times 10^{-14}$ m·s${}^{-1}$ and 0.41 eV for $D_{0}^{\mathrm{ox}}$ and $e_{m}^{\mathrm{ox}}$, respectively. Alloy composition, however, has been shown to have a significant impact on diffusion parameters. For example, values of $e_{m}^{\mathrm{ox}}$ = 0.55 and 1.0 eV have been reported for Zr-2.5%Nb and pure Zr, respectively, with $D_{0}$ numbers in as high as $1.1\times 10^{-12}$ m·s${}^{-1}$ [81,82]. Here, we use the parameters for Zirc-4 given by Tupin et al.

Similarly, the only mobile species in the metal is monoatomic hydrogen. The most widely used parameters for hydrogen diffusion in metal Zr, and Zircaloy-2 and -4 ($D_{i}$ in Equation (12)) are those by Kearns [83] in the 200–700 ${}^{\circ }$C temperature range, with values of $D_{0}^{m}=7.90\times 10^{-7}$ m·s${}^{-1}$ and $e_{m}^{m}=0.46$ eV. Earlier literature on these measurements [25,84,85] reveals pre-factors ranging from $7.00\times 10^{-8}$ to $4.15\times 10^{-7}$ m·s${}^{-1}$ and migration energies between 0.3 and 0.5 eV, all in a similar temperature range. More recent experiments and molecular dynamics simulations are also consistent with these values [86,87].

The values chosen here for each case (diffusion in the oxide and in the metal) are given un Table 1.

#### 2.3.2. Nucleation of Zr${}_{2}$H${}_{3}$ Hydride

As shown in Figure 2, once hydrogen penetrates into the metal clad, the hydration reaction $\mathrm{Zr}+xH\to {\mathrm{ZrH}}_{x}$ starts occurring. Although the formation of the δ-hydride is seen for a range of *x* values, here we assume a perfect stoichiometry of *x*=1.5. Consequently, the governing equilibrium constant for the reaction can be expressed as:

$$K_{\delta }=\frac{\left[{\mathrm{ZrH}}_{1.5}\right]}{\left[\mathrm{Zr}\right]{\left[H\right]}^{1.5}}$$

**Figure 4:**
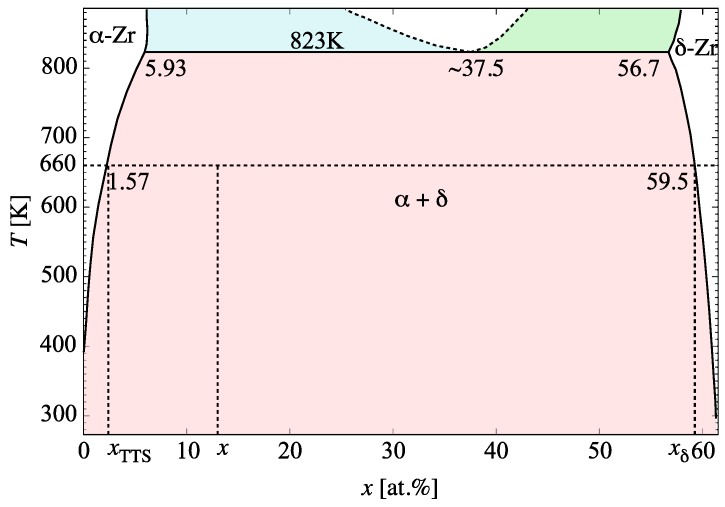
Phase diagram of the Zr-H system in the temperature and concentration region relevant to the present study (adapted from several sources [21,89,90]).

However, it is more convenient to use an expression that is linear in the hydrogen concentration. From this, one can write the reaction rate as:

(14) $$k_{\delta }=4\pi \left(r_{H}+r_{\mathrm{Zr}}\right)\left(V^{-\frac{1}{3}}\rho_{\mathrm{Zr}}^{\frac{2}{3}}\right)D_{H}N_{H}p\left(x\right)\exp\left(-\frac{\Delta E_{\delta }}{kT}\right)$$

which is simply a coagulation rate for two species –H and Zr– in the proportions indicated by the exponents of $\rho_{\mathrm{Zr}}$ and $N_{H}$. $\Delta E_{\delta }$ is the formation energy of a molecule of δ hydride (≈0.52 eV at 350 ${}^{\circ }$C according to Blomqvist et al. [88]) and p(x) represents the thermodynamic probability for this reaction to occur, which can be directly extracted from the Zr-H phase diagram using the lever rule:

(15) $$p\left(x\right)=\frac{x-x_{\mathrm{TTS}}}{x_{\delta }-x_{\mathrm{TTS}}}$$

where $x_{\mathrm{TTS}}$ is the *terminal thermal solubility* at the temperature of interest, and $x_{\delta }$ is the phase boundary. A phase diagram of the Zr-H system in the temperature and concentration region relevant to the present study is shown in Figure 4. By way of example, at 660 K (horizontal dashed line in the figure) $x_{\mathrm{TTS}}$ is approximately 1.6% at. and $x_{\delta }$≈60.0% at.

Equation (15) ensures that $p(x_{\mathrm{TTS}})=0$ and $p(x_{\delta })=1$, i.e. the nucleation probability is zero at the phase boundary between the (α) and (α+δ) regions, and unity at the (α+δ) and (δ) boundary. This simple factor captures the thermodynamic propensity for the hydride reaction to take place, and thus ties the thermodynamics and kinetics of hydration together. $r_{H}$ and $r_{\mathrm{Zr}}$ in Equation (14) are the interaction radii of H and Zr, respectively, their values given in Table 2. In the SCD calculations, the atomic fraction *x* is simply defined at any instant in time as:

$$x=\frac{N_{H}}{N_{H}+\rho_{\mathrm{Zr}}V}$$

#### 2.3.3. Growth of Zr${}_{2}$H${}_{3}$ Hydride

Once hydride nuclei appear in the clad, their growth is treated as a standard coagulation process in 3D with rate constant:

(16) $$k_{n}=4\pi V^{-1}\left(r_{H}+r_{\delta }\left(n\right)\right)D_{H}N_{H}N_{\left({\mathrm{Zr}}_{0.66n}H_{n}\right)}\exp\left(-\frac{\Delta E_{\delta }}{kT}\right)$$

where $r_{\delta }$ is the interaction radius of the hydride clusters, and $N_{\left({\mathrm{Zr}}_{0.66n}H_{n}\right)}$ is the concentration of a hydride cluster containing *n* H atoms (which implies having 0.66n Zr atoms). It is assumed that hydride clusters are immobile. In accordance with previous works, hydrides grow as circular platelets whose size is directly related to the number of hydrogen monomers contained in it [56]:

$$r_{\delta }\left(n\right)=\sqrt{\frac{n\Omega_{H}}{\pi d}}$$

with $\Omega_{H}$ and *d* the formation volume of hydrogen and the thickness of the platelet, respectively. As given in Table 2, here we use $\Omega_{H}=2.8\times 10^{-3}$ nm${}^{3}$ per atom [91], and d≈0.28 nm [37].

The growth of hydride platelets is known to be highly directional, and influenced by stress and microstructure. Typically hydrides align themselves along the direction of the dominant axial stress components and grow preferentially in-plane on grain boundaries [27,36,37,53]. These details are not captured in our model at present.

#### 2.3.4. Dissolution of Zr${}_{2}$H${}_{3}$ Hydride

The last process considered in our model is the thermal dissolution of the hydrides, as Figure 4 shows, strictly speaking, hydrides are stable up to 550 ${}^{\circ }$C (eutectoid temperature), although there is ample evidence of their decomposition at much lower temperatures, as well as the observation of thermal hysteresis during heating/cooling cycles [37,92,93,94]. The dissociation rate is a first-order process that can be expressed as:

(17) $$s_{n}=4\pi r_{\mathrm{ffi}}\left(n\right)D_{H}N_{\left({\mathrm{Zr}}_{0.66n}H_{n}\right)}\exp\{(-\frac{e_{b}\left(n\right)}{kT})\}$$

where $e_{b}$ is the binding energy between a H monomer and a cluster containing *n* hydrogen atoms. Here, we assume a capillary approximation for $e_{b}$ [55,56]:

$$e_{b}\left(n\right)=e_{s}-0.44\left[n^{\frac{2}{3}}-{\left(n-1\right)}^{\frac{2}{3}}\right]$$

with $e_{s}$ being the heat of solution of H in the α-Zr matrix. This parameter has been found to be approximately 0.45 eV in electronic structure calculations [95,96], compared to 0.66 eV in experiments [97].

#### 2.3.5. Metal/Oxide Interface Motion

Finally, the motion of the interface must also be considered as a viable stochastic event. To turn the interface velocity, Equation (5), into an event rate, $r_{i}$, one simply normalizes it by the interface thickness, *s*.

$$r_{i}=\left(\frac{1}{s}\right)\frac{ds}{dt}=\frac{ant^{n-1}}{at^{n}}=\frac{n}{t}$$

which results in the following expression for $r_{i}$:

(18) $$r_{i}=0.34t^{-1}$$

This is added to the event catalog and sampled with the corresponding probability as given by Equation (18). As the equation shows, this is a time-dependent rate that reflects the nonlinear growth of the oxide layer with time. In the context of the SCD model, it implies that the one-dimensional mesh shown in Figure 3 must be dynamically updated with time because the physical dimensions of the simulation domain are dynamically changed. To our knowledge, this has not been attempted in any prior models of hydride formation and buildup.

### 2.4. Parameterization, Physical Dimensions, and Boundary Conditions

All the material constants used in the present model are given in Table 1 and Table 2. External parameters representing the geometry and the boundary conditions are given in Table 3.

## 3. Results

The first two figures show results intended to set the stage for the SCD calculations.

**Figure 5:**
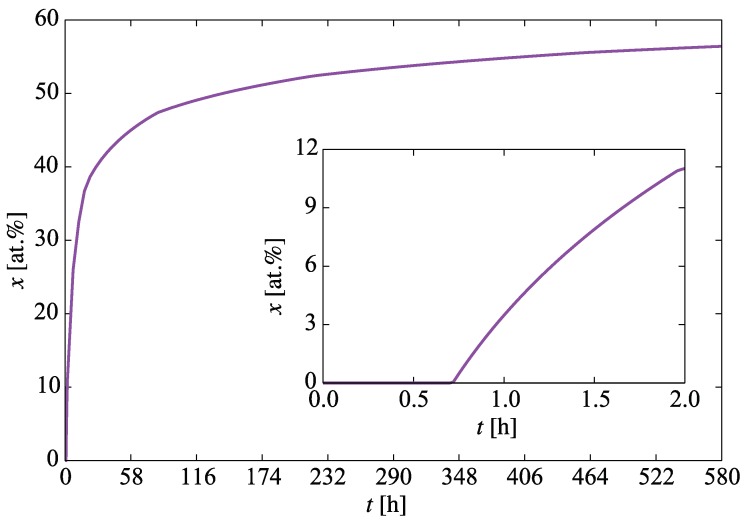
Evolution with time of the hydrogen concentration at the oxide/metal interface. This represents the boundary condition for the spatially-resolved SCD calculations of hydride nucleation and buildup.

**Figure 6:**
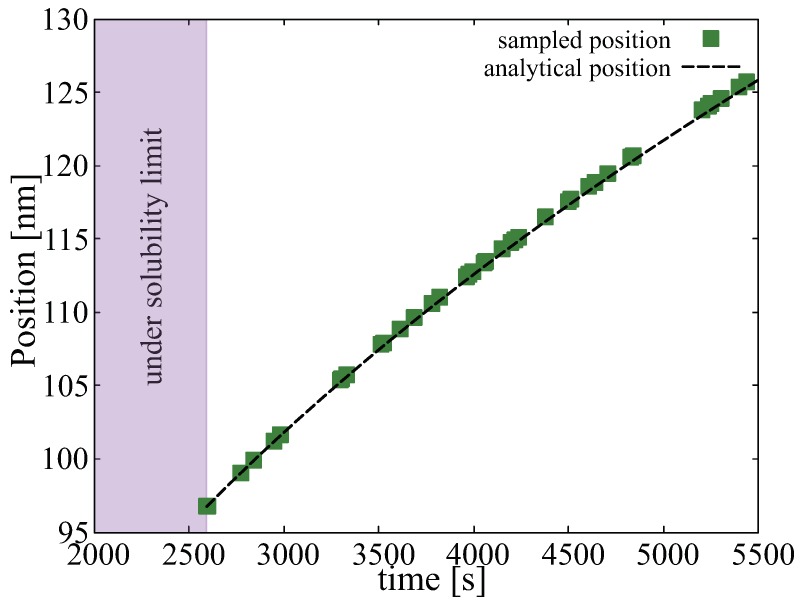
Comparison between the predictions of $r_{i}$ and Equation (18) of the position of the oxide/metal interface as a function of time. We track the interface position only after the concentration of hydrogen has reached the solubility limit.

We begin with the time evolution of the hydrogen concentration at the oxide/metal interface. This results from solving the diffusion equation in the the oxide layer subjected to a moving boundary as explained in Section 2.2. Figure 5 shows the buildup of hydrogen up to the first 580 h. This represents a *dynamic* Dirichlet boundary condition for the spatially-resolved SCD calculations of hydride nucleation and growth in the metal substrate ($\tilde{g}$ term in Equation (12)). Second, we track the sampling rate $r_{i}$ defined in Section 2.3.5 to confirm that it matches Equation (18). Figure 6 shows a comparison between both, indeed demonstrating their equivalency and confirming the correctness of its implementation in the code. The effect of this interface motion is that, over the course of the time scale covered in the SCD simulations, the oxide layer effectively sweeps over the first mesh element of the metal depth profile (recall that we assume that such sweep results in dissolution of the hydrides existing within that element at that point, and re-solution of the immobilized hydrogen in the metal). In practice, this allows us to subsequently discard the first spatial element of the 1D mesh. That is the reason why in the figures shown next the spatial range shown spans 800 (as opposed to the original 900) nm.

**Figure 7:**
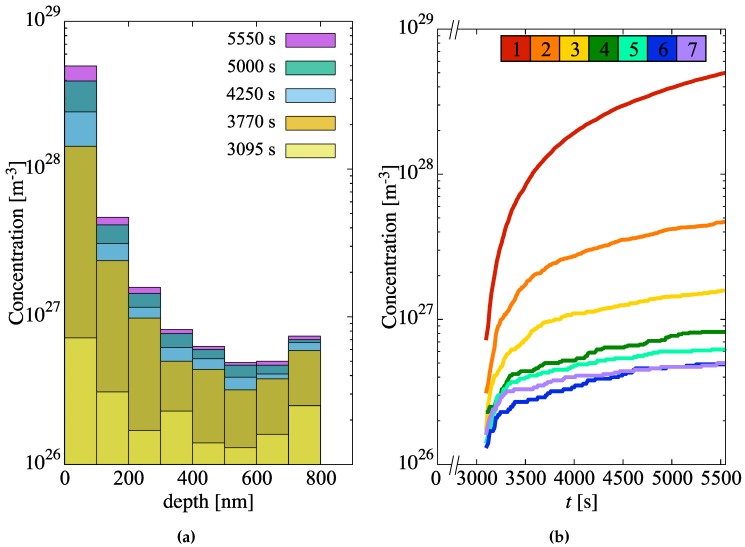
(**a**) Concentration of incipient hydride nuclei in the metal layer as a function of depth for several time snapshots. (**b**) Hydride concentration buildup as a function of time for each depth element. Each curve is colored according to the key at the top of the figure (element 1 is closest to the oxide/metal interface). Per Table 3, each element is 100-nm thick.

Next, we study the generation of hydride molecules in the metal layer as a function of time and depth. The results are shown in Figure 7a, which shows a histogram with the concentration of hydride molecules at several instants in time for each of the mesh elements of the metal region. As discussed in Section 2.3.2, the probability that a new hydride molecule will form depends primarily on the relative H concentration at the interface and the heat of formation of δ-hydride. With a probability per unit time $k_{\delta }$ (Equation (14)), freely-diffusing H atoms are immobilized to form Zr${}_{2\;/\;3}$H molecules that act as incipient hydride nuclei. The concentrations of such nuclei are strongly depth-dependent, as shown in the figure, ranging over two orders of magnitude over the entire specimen thickness *L* of 900 nm. As well, the nucleation rate, i.e., the derivative of the evolution curves shown in Figure 7b (which display the same data as Figure 7a but plotted as a function of time), can be seen to decrease gradually in time across the entire depth profile.

**Figure 8:**
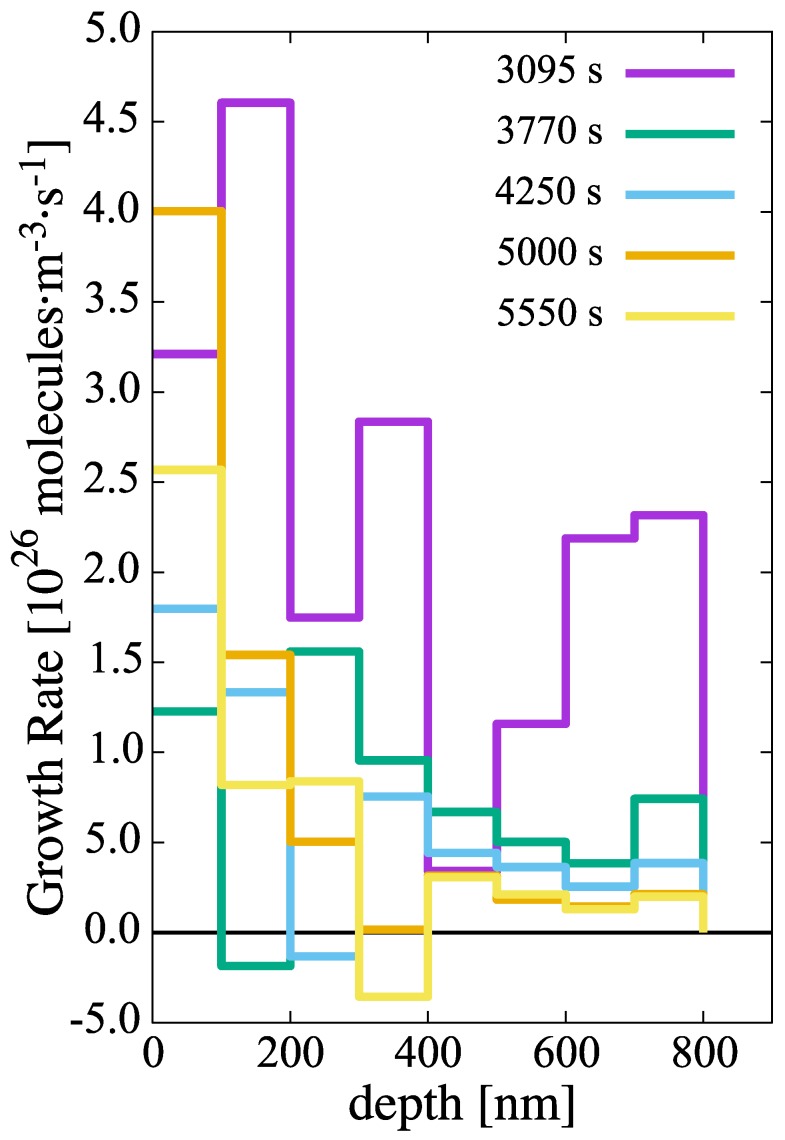
Growth rate of hydride clusters in the metal layer as a function of time and depth.

Subsequent growth of these embryos occurs at a rate given by the combination of the rates of H-atom absorption (Equation (16)) and dissolution (Equation (17)), i.e., $\left(k_{n}-s_{n}\right)$, as shown in Figure 8. Rapid net growth is seen in the initial stages of hydridization close to the oxide/metal interface. However, these rates gradually abate both in time and with increasing depth until almost no net growth is observed, particularly at depths greater than 700 nm after 1.4 h of evolution.

**Figure 9:**
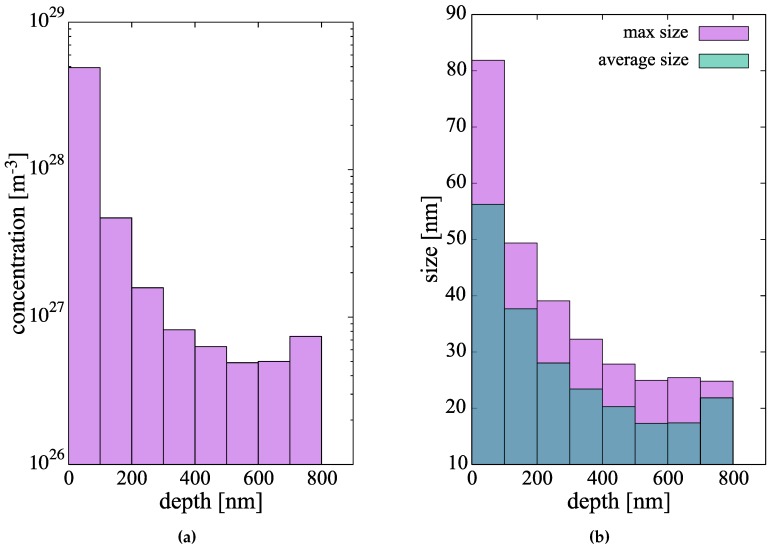
(**a**) Concentration profile and (**b**) size distributions of the hydride cluster population after 1.53 h of simulated evolution.

The resulting hydride concentrations across the 900-nm metal layer at the end of the simulated time can be found in Figure 9a. As the graph indicates, the hydride number densities suffer almost a 100-fold decrease through the metal layer studied. In relative terms, these are large concentrations of small clusters, so it is to be expected that further time evolution of the hydride subpopulations will be dominated by growth, perhaps by way of some type of coarsening or ripening mechanism. The associated size distributions of the hydride clusters are shown in Figure 9b, where both the average and maximum cluster sizes are shown. We emphasize that, during the incipient nucleation of the hydrides, they grow as circular discs, and so the sizes simulated (≈50 nm or less), correspond to the regime prior to the acicular growth of the hydrides.

## 4. Discussion

Several of the most important features of the model presented here are: (i) consideration of a moving interface representing the growth of the oxide scale during operation in corrosive conditions; (ii) using a hydride nucleation criterion that is consistent with the thermodynamics of the Zr-H system; (iii) using a mean-field growth/dissolution model that respects; (iv) a completely physics-based parameterization based on calculated atomistic data. Some of these features were part of a comparable study [56], to which new ones have been added and existing ones augmented. All these features combined are the basis of a model that has been developed as an attempt to break the phenomenological vicious cycle in which models of materials degradation in nuclear environments are often found.

To study the nucleation of the hydride clusters, our method samples discrete kinetic processes defined by the corresponding energetics and thermodynamics. For example, hydride nucleation is simulated by considering the interplay between (i) aggregation, (ii) growth, and (iii) dissolution processes, which together determine the net nucleation and growth rates. Processes (i), (ii), and (iii) are embodied in Equations (14), (16) and (17), respectively. Each one of these processes is treated as a stochastic event sampled with the probabilities given by each respective rate. If the conditions are such that dissolution would dominate over nucleation, the clusters would never form. If growth dominates over nucleation, the clusters would grow bigger, etc. All the energetics are given by the parameters in each of those equations.

As is often the case, the price paid for an increased physical fidelity in the simulations is computational efficiency. For this reason, our simulations can only extend to times of several thousand seconds (<2 h), which is of course only representative of the initial stages of hydridation in Zr clad (and, of course, part of the pre-transition corrosion regime)). In these relatively short time scales (which are still orders of magnitude higher than what direct atomistic methods can cover), one can only claim to faithfully study the incipient nucleation phase of the hydride microstructure. In this sense, our results do not include important features of the Zr hydride particles such as their elongated shape and/or their orientation. Excellent recent examples of experimental characterization displaying all of these structure complexities exist now in the literature [99,100,101]. They can act, however, as a good springboard from which to connect to other methods such as phase field simulations [27,102,103], or orientation-dependent precipitation models [104,105]. Therefore, it is reasonable to assume that the time scale of the next phase of hydride formation/growth kinetics would be one dominated by coarsening/ripening, where population densities suffer a gradual decline at the expense of an increased average precipitate size. Thus, it is important to emphasize this aspect of the work: our results correspond to the incipient hydride nucleation and growth phase, before steady state populations are established. Steady state sizes and concentrations in corroded Zr specimens range from 100 nm to 1 μm [101] and ∼$10^{24}$ m${}^{-3}$. On this aspect, it is also difficult to reconcile calculated H-atom diffusivities in the clad with almost cross-clad uniform hydride distributions observed experimentally [106]. Calculated migration energies suggest a much more sluggish diffusion in the metal, and screening of the clad interior by hydrides formed near the oxide metal interface, as seen in this study, compared to experimental results. While validation on the time and length scales covered in this work is always difficult, it is encouraging to see reasonable qualitative agreement with experimental studies, e.g., hydride precipitation completion fractions in ref. [36] (Figure 3) vs. Figure 7b in this paper. As well, our predictions for the size (long axis) of the precipitates in Figure 9b are in good agreement with *in situ* SEM observations [107].

As reviewed in the Introduction section, the formation of Zr hydrides in the metal clad is considered to be highly detrimental to reactor performance due to their embrittling effect. However, the high thermal stability of these hydride phases also makes them a matter of concern for reactor safety due to the potential for hydrogen storage and release during loss-of-coolant conditions and core meltdown. Palliative measures such as increasing the enthalpy of formation of δ-ZrH by microstructure tailoring [108], or by hindering H diffusion in Zr oxide by selective alloying in the clad [109,110], have been proposed for future candidate materials in novel nuclear fuel designs.

## 5. Conclusions

We end this paper with a list of the most important conclusions:

- We have developed a spatially-resolved kinetic model of hydrogen transport/accumulation in Zr-metal clad. The model includes state-of-the-art hydride energetics data from atomistic calculations and is formulated as a stochastic version of the cluster dynamics method. Notably, boundary conditions are dynamically updated in time during the simulations, by accounting for oxide/metal interface motion due to the time-dependent growth of the oxide scale.
- In doing so, our model is consistent with the oxidation in the clad, as well as with the equilibrium thermodynamics of the Zr-H system.
- As most cluster dynamics models based on mean-field rate theory, our model does not capture the orientation dependence of elongated hydride platelets observed experimentally, and microstructural information such as grain sizes and dislocation densities is included only in an effective way. As such, our results are representative of the ‘average’ structure along the depth direction.
- Our results show that high concentrations of small hydride nuclei form across the entire metal clad. This results in a very fine microstructure that sets the stage for the next kinetic phase, likely to be one of ripening and coarsening.
- Gaps in our knowledge identified in this work include, among others: (i) how to model the H dissolved from hydrides swept by the growing oxide layer, (ii) how to reconcile existing H-atom diffusion energies with almost cross-clad uniform hydride distributions, and (iii) the reasons for the acicular (or capsular) growth of the precipitates are still not clear and, while such geometries can be adopted in the models, a physical approach that yields these geometric features is still lacking.

## Figures and Tables

**Table 1:** Zr-H energetics used in the model with the respective source.

Parameter	Unit	Symbol	Value	Source
Hydrogen diffusivity prefactor in Zr oxide	m·s${}^{-1}$	$D_{H}^{\mathrm{ox}}$	$2.50\times 10^{-14}$	[80]
Hydrogen migration energy in Zr oxide	eV	$e_{m}^{\mathrm{ox}}$	0.41	[80]
Hydrogen diffusivity prefactor in Zr metal	m·s${}^{-1}$	$D_{H}^{m}$	$7.90\times 10^{-7}$	[83]
Hydrogen migration energy in Zr metal	eV	$e_{m}^{m}$	0.46	[83]
δ-hydride formation energy	eV	$\Delta E_{\delta }$	0.88	[88]
H solution energy in Zr metal	eV	$e_{s}$	0.66	[97]

**Table 2:** Physical constants for the Zr-H system employed here. In actuality, the interaction radii of Zr and H atoms are extended by a distance equal to the Burgers vector 〈a〉 in α-Zr, which is equal to 3.23 Å.

Physical Constant	Symbol	Unit	Value	Source
Zr atomic density	$\rho_{\mathrm{Zr}}$	m${}^{-3}$	$4.31\times 10^{28}$	-
H-atom interaction radius	$r_{H}$	Å	0.31	[98]
Zr-atom interaction radius	$r_{\mathrm{Zr}}$	Å	1.75	[98]
H-atom formation volume	$\Omega_{H}$	nm${}^{3}$ per atom	$2.8\times 10^{-3}$	[91]
δ-hydride platelet thickness	*d*	nm	0.28	[37]

**Table 3:** Numerical parameters used in the model.

$f_{H}$	$x_{\mathrm{TTS}}$ [%]	$x_{\delta }$ [%]	*T* [K]	*V* $\left[m^{-3}\right]$	*l* [nm]	*L* [nm]
0.15	1.6	59.5	660	$10^{-18}$	100	900

## References

[1] Scully J.C. (1978). The Fundamentals of Corrosion.

[2] North N., MacLeod I.D., Pearson C. (1987). Corrosion of Metals.

[3] Young D.J. (2008). High Temperature Oxidation and Corrosion of Metals.

[4] Comstock R., Motta A.T. (2018). Zirconium in the Nuclear Industry: 18th International Symposium.

[5] Clayton J.C. (1984). Out-of-pile nickel alloy-induced accelerated hydriding of zircaloy fasteners. Zirconium in the Nuclear Industry.

[6] Jacques P., Lefebvre F., Lemaignan C. (1999). Deformation–corrosion interactions for Zr alloys during I-SCC crack initiation: part I: chemical contributions. J. Nucl. Mater..

[7] Sabol G.P., Moan G.D. (2000). Zirconium in the Nuclear Industry: Twelfth International Symposium.

[8] Féron D. (2012). Nuclear Corrosion Science and Engineering.

[9] Cattant F., Crusset D., Féron D. (2008). Corrosion issues in nuclear industry today. Mater. Today.

[10] Allen T., Konings R., Motta A. (2012). 5.03 corrosion of zirconium alloys. Compr. Nucl. Mater..

[11] Preuss M. Zirconium cladding-the long way towards a mechanistic understanding of processing and performance. Proceedings of the Second International Conference on Advances in Nuclear Materials: Abstract Booklet and Souvenir.

[12] Hillner E. (1977). Corrosion of zirconium-base alloys? An overview. Zirconium in the Nuclear Industry.

[13] Zaimovskii A. (1978). Zirconium alloys in nuclear power. At. Energy.

[14] Cox B. (2005). Some thoughts on the mechanisms of in-reactor corrosion of zirconium alloys. J. Nucl. Mater..

[15] Causey R.A., Cowgill D.F., Nilson R.H. (2005). Review of the Oxidation Rate of Zirconium Alloys.

[16] Motta A.T., Yilmazbayhan A., da Silva M.J.G., Comstock R.J., Was G.S., Busby J.T., Gartner E., Peng Q., Jeong Y.H., Park J.Y. (2007). Zirconium alloys for supercritical water reactor applications: Challenges and possibilities. J. Nucl. Mater..

[17] Bossis P., Pecheur D., Hanifi K., Thomazet J., Blat M. (2006). Comparison of the high burn-up corrosion on M5 and low tin Zircaloy-4. 14th International Symposium on Zirconium in the Nuclear Industry.

[18] Motta A.T., Capolungo L., Chen L.Q., Cinbiz M.N., Daymond M.R., Koss D.A., Lacroix E., Pastore G., Simon P.C.A., Tonks M.R. (2019). Hydrogen in zirconium alloys: A review. J. Nucl. Mater..

[19] McRae G., Coleman C., Leitch B. (2010). The first step for delayed hydride cracking in zirconium alloys. J. Nucl. Mater..

[20] Zieliński A., Sobieszczyk S. (2011). Hydrogen-enhanced degradation and oxide effects in zirconium alloys for nuclear applications. Int. J. Hydrog. Energy.

[21] Zuzek E., Abriata J., San-Martin A., Manchester F. (1990). The H-Zr (hydrogen-zirconium) system. Bull. Alloy Phase Diagr..

[22] Dupin N., Ansara I., Servant C., Toffolon C., Lemaignan C., Brachet J. (1999). A thermodynamic database for zirconium alloys. J. Nucl. Mater..

[23] Steinbrück M. (2004). Hydrogen absorption by zirconium alloys at high temperatures. J. Nucl. Mater..

[24] Grosse M., Steinbrueck M., Lehmann E., Vontobel P. (2008). Kinetics of Hydrogen Absorption and Release in Zirconium Alloys During Steam Oxidation. Oxid. Met..

[25] Gulbransen E.A., Andrew K.F. (1954). Diffusion of hydrogen and deuterium in high purity zirconium. J. Electrochem. Soc..

[26] Root J., Small W., Khatamian D., Woo O. (2003). Kinetics of the *δ* to *γ* zirconium hydride transformation in Zr-2.5Nb. Acta Mater..

[27] Zhao Z., Blat-Yrieix M., Morniroli J., Legris A., Thuinet L., Kihn Y., Ambard A., Legras L. (2009). Characterization of zirconium hydrides and phase field approach to a mesoscopic-scale modeling of their precipitation. Zirconium in the Nuclear Industry: 15th International Symposium.

[28] Ackland G. (1998). Embrittlement and the bistable crystal structure of zirconium hydride. Phys. Rev. Lett..

[29] Olsson P., Massih A., Blomqvist J., Holston A.M.A., Bjerkén C. (2014). Ab initio thermodynamics of zirconium hydrides and deuterides. Comput. Mater. Sci..

[30] Zhu W., Wang R., Shu G., Wu P., Xiao H. (2010). First-principles study of different polymorphs of crystalline zirconium hydride. J. Phys. Chem. C.

[31] Chernov I.I., Staltsov M.S., Kalin B.A., Guseva L.Y. (2017). Some problems of hydrogen in reactor structural materials: A review. Inorg. Mater. Appl. Res..

[32] Coleman C., Hardie D. (1966). The hydrogen embrittlement of *α*-zirconium? A review. J. Less Common Met..

[33] Tummala H., Capolungo L., Tome C.N. (2017). Quantifying the Stress Fields Due to a Delta-Hydride Precipitate in Alpha-Zr Matrix.

[34] Bloch J. (1995). The temperature-dependent changes of the kinetics and morphology of hydride formation in zirconium. J. Alloys Compd..

[35] Motta A.T., Chen L.Q. (2012). Hydride formation in zirconium alloys. JOM.

[36] Blackmur M.S., Robson J., Preuss M., Zanellato O., Cernik R., Shi S.Q., Ribeiro F., Andrieux J. (2015). Zirconium hydride precipitation kinetics in Zircaloy-4 observed with synchrotron X-ray diffraction. J. Nucl. Mater..

[37] Cinbiz M.N., Koss D.A., Motta A.T., Park J.S., Almer J.D. (2017). In situ synchrotron X-ray diffraction study of hydrides in Zircaloy-4 during thermomechanical cycling. J. Nucl. Mater..

[38] Ells C. (1968). Hydride precipitates in zirconium alloys (A review). J. Nucl. Mater..

[39] Carpenter G. (1973). The dilatational misfit of zirconium hydrides precipitated in zirconium. J. Nucl. Mater..

[40] Singh R.N., Ståhle P., Massih A.R., Shmakov A. (2007). Temperature dependence of misfit strains of *δ*-hydrides of zirconium. J. Alloys Comp..

[41] Barrow A., Korinek A., Daymond M. (2013). Evaluating zirconium–zirconium hydride interfacial strains by nano-beam electron diffraction. J. Nucl. Mater..

[42] Lumley S., Grimes R., Murphy S., Burr P., Chroneos A., Chard-Tuckey P., Wenman M. (2014). The thermodynamics of hydride precipitation: The importance of entropy, enthalpy and disorder. Acta Mater..

[43] Chan K.S. (2013). An assessment of delayed hydride cracking in zirconium alloy cladding tubes under stress transients. Int. Mater. Rev..

[44] Markelov V.A. (2011). Delayed hydride cracking of zirconium alloys: Appearance conditions and basic laws. Russ. Metall. (Met.).

[45] Lee H., min Kim K., Kim J.S., Kim Y.S. (2019). Effects of hydride precipitation on the mechanical property of cold worked zirconium alloys in fully recrystallized condition. Nucl. Eng. Technol..

[46] Likhanskii V., Evdokimov I. Review of theoretical conceptions on regimes of oxidation and hydrogen pickup in Zr-alloys. Proceedings of the International Conference on WWER Fuel Performance, Modelling and Experimental Eupport.

[47] Steinbrück M., Birchley J., Goryachev A., Grosse M., Haste T., Hozer Z., Kisselev A., Nalivaev V., Semishkin V., Sepold L. Status of studies on high-temperature oxidation and quench behaviour of Zircaloy-4 and E110 cladding alloys. Proceedings of the 3rd European Review Meeting on Severe Accident Research (ERMSAR-2008).

[48] Lindgren M., Panas I. (2014). On the fate of hydrogen during zirconium oxidation by water: effect of oxygen dissolution in [small alpha]-Zr. RSC Adv..

[49] Chen W., Wang L., Lu S. (2009). Influence of oxide layer on hydrogen desorption from zirconium hydride. J. Alloys Comp..

[50] Couet A., Motta A.T., Ambard A., Comstock R. Oxide electronic conductivity and hydrogen pickup fraction in Zr alloys. Proceedings of the 2014 Annual Meeting on Transactions of the American Nuclear Society and Embedded Topical Meeting: Nuclear Fuels and Structural Materials for the Next Generation Nuclear Reactors, NSFM.

[51] Puls M.P. (2009). Review of the thermodynamic basis for models of delayed hydride cracking rate in zirconium alloys. J. Nucl. Mater..

[52] Marino G. (1971). Hydrogen supercharging in Zircaloy. Mater. Sci. Eng..

[53] Tikare V. (2013). Simulation of Hydride Reorientation in Zr-Based Claddings During Dry Storage.

[54] Courty O., Motta A.T., Hales J.D. (2014). Modeling and simulation of hydrogen behavior in Zircaloy-4 fuel cladding. J. Nucl. Mater..

[55] Aryanfar A., Thomas J., Van der Ven A., Xu D., Youssef M., Yang J., Yildiz B., Marian J. (2016). Integrated computational modeling of water side corrosion in zirconium metal clad under nominal LWR operating conditions. JOM.

[56] Xu D., Xiao H., Jackson J.H., Paraventi D., Wright M. (2019). Cluster Dynamics Model for the Hydride Precipitation Kinetics in Zirconium Cladding. Proceedings of the 18th International Conference on Environmental Degradation of Materials in Nuclear Power Systems–Water Reactors.

[57] Ma X., Shi S., Woo C., Chen L. (2006). The phase field model for hydrogen diffusion and *γ*-hydride precipitation in zirconium under non-uniformly applied stress. Mech. Mater..

[58] Guo X., Shi S., Zhang Q., Ma X. (2008). An elastoplastic phase-field model for the evolution of hydride precipitation in zirconium. Part I: Smooth specimen. J. Nucl. Mater..

[59] Aryanfar A., Goddard W., Marian J. (2019). Constriction Percolation Model for Coupled Diffusion-Reaction Corrosion of Zirconium in PWR. Corros. Sci..

[60] Une K. (1978). Kinetics of reaction of Zirconium alloy with hydrogen. J. Less Common Met..

[61] Wang X., Zheng M.J., Szlufarska I., Morgan D. (2017). Continuum model for hydrogen pickup in zirconium alloys of LWR fuel cladding. J. Appl. Phys..

[62] Lim B.H., Hong H.S., Lee K.S. (2003). Measurements of hydrogen permeation and absorption in zirconium oxide scales. J. Nucl. Mater..

[63] Geelhood K., Beyer C. Hydrogen Pickup Models for Zircaloy-2, Zircaloy-4, M5^TM^, and ZIRL^TM^. Proceedings of the 2011 Water Reactor Fuel Performance Meeting.

[64] Couet A., Motta A.T., Comstock R.J. (2014). Hydrogen pickup measurements in zirconium alloys: Relation to oxidation kinetics. J. Nucl. Mater..

[65] Chernyayeva T.P., Ostapov A. (2013). Hydrogen in zirconium part 1. Probl. At. Sci. Technol..

[66] Hu J., Liu J., Lozano-Perez S., Grovenor C.R., Christensen M., Wolf W., Wimmer E., Mader E.V. (2019). Hydrogen pickup during oxidation in aqueous environments: The role of nano-pores and nano-pipes in zirconium oxide films. Acta Mater..

[67] Heuser B.J., Lin J.L., Do C., He L. (2018). Small-angle neutron scattering measurements of *δ*-phase deuteride (hydride) precipitates in Zircaloy 4. J. Appl. Crystallogr..

[68] Zhao Z., Morniroli J.P., Legris A., Ambard A., Khin Y., Legras L., Blat-Yrieix M. (2008). Identification and characterization of a new zirconium hydride. J. Microsc..

[69] Reyes M., Aryanfar A., Baek S.W., Marian J. (2018). Multilayer interface tracking model of zirconium clad oxidation. J. Nucl. Mater..

[70] Garzarolli F., Seidel H., Tricot R., Gros J. (1991). Oxide growth mechanism on zirconium alloys. Zirconium in the Nuclear Industry: Ninth International Symposium.

[71] Billot P., Cox B., Ishigure K., Johnson A., Lemaignan C., Nechaev A., Petrik N., Reznichenko E., Ritchie I.G., Sukhanov G.I. (1993). Corrosion of Zirconium Alloys in Nuclear Power Plants.

[72] Motta A.T., Couet A., Comstock R.J. (2015). Corrosion of Zirconium Alloys Used for Nuclear Fuel Cladding. Ann. Rev. Mater. Res..

[73] Chevalier J., Gremillard L., Virkar A.V., Clarke D.R. (2009). The Tetragonal-Monoclinic Transformation in Zirconia: Lessons Learned and Future Trends. J. Am. Ceram. Soc..

[74] Whitney E.D. (1965). Kinetics and mechanism of the transition of metastable tetragonal to monoclinic zirconia. Trans. Faraday Soc..

[75] Couet A., Motta A.T., Ambard A. (2015). The coupled current charge compensation model for zirconium alloy fuel cladding oxidation: I. Parabolic oxidation of zirconium alloys. Corros. Sci..

[76] Marian J., Bulatov V.V. (2011). Stochastic cluster dynamics method for simulations of multispecies irradiation damage accumulation. J. Nucl. Mater..

[77] Marian J., Hoang T.L. (2012). Modeling fast neutron irradiation damage accumulation in tungsten. J. Nucl. Mater..

[78] Dunn A.Y., Capolungo L., Martinez E., Cherkaoui M. (2013). Spatially resolved stochastic cluster dynamics for radiation damage evolution in nanostructured metals. J. Nucl. Mater..

[79] Dunn A., Capolungo L. (2015). Simulating radiation damage accumulation in *α*-Fe: a spatially resolved stochastic cluster dynamics approach. Comput. Mater. Sci..

[80] Tupin M., Martin F., Bisor C., Verlet R., Bossis P., Chêne J., Jomard F., Berger P., Pascal S., Nuns N. (2017). Hydrogen diffusion process in the oxides formed on zirconium alloys during corrosion in pressurized water reactor conditions. Corros. Sci..

[81] Cox B. (1985). Mechanisms of Hydrogen Absorption by Zirconium Alloys.

[82] Khatamian D., Manchester F. (1989). An ion beam study of hydrogen diffusion in oxides of Zr and Zr-Nb (2.5 wt%): I. Diffusion parameters for dense oxide. J. Nucl. Mater..

[83] Kearns J. (1972). Diffusion coefficient of hydrogen in alpha zirconium, Zircaloy-2 and Zircaloy-4. J. Nucl. Mater..

[84] Sawatzky A. (1960). The diffusion and solubility of hydrogen in the alpha phase of Zircaloy-2. J. Nucl. Mater..

[85] Someno M. (1960). Solubility and diffusion of hydrogen in zirconium. Nippon Kinzoku Gakkaishi Jpn..

[86] Grosse M., Van Den Berg M., Goulet C., Kaestner A. (2012). In-situ investigation of hydrogen diffusion in Zircaloy-4 by means of neutron radiography. J. Phys. Conf. Ser..

[87] Siripurapu R.K., Szpunar B., Szpunar J.A. (2014). Molecular Dynamics Study of Hydrogen in *α*-Zirconium. Int. J. Nucl. Energy.

[88] Blomqvist J., Olofsson J., Alvarez A.M., Bjerkén C., Busby J.T., Ilevbare G., Andresen P.L. (2016). Structure and Thermodynamical Properties of Zirconium Hydrides from First-Principle. Proceedings of the 15th International Conference on Environmental Degradation of Materials in Nuclear Power Systems—Water Reactors.

[89] Miyake M., Uno M., Yamanaka S. (1999). On the zirconium–oxygen–hydrogen ternary system. J. Nucl. Mater..

[90] LaGrange L.D., Dykstra L., Dixon J.M., Merten U. (1959). A Study of the Zirconium-Hydrogen and Zirconium-Hydrogen–Uranium Systems between 600 and 800^∘^. J. Phys. Chem..

[91] Weekes H., Dye D., Proctor J., Smith D., Simionescu C., Prior T., Wenman M. (2018). The Effect of Pressure on Hydrogen Solubility in Zircaloy-4. arXiv.

[92] Northwood D., Kosasih U. (1983). Hydrides and delayed hydrogen cracking in zirconium and its alloys. Int. Met. Rev..

[93] Une K., Ishimoto S. (2003). Dissolution and precipitation behavior of hydrides in Zircaloy-2 and high Fe Zircaloy. J. Nucl. Mater..

[94] Zanellato O., Preuss M., Buffiere J.Y., Ribeiro F., Steuwer A., Desquines J., Andrieux J., Krebs B. (2012). Synchrotron diffraction study of dissolution and precipitation kinetics of hydrides in Zircaloy-4. J. Nucl. Mater..

[95] Domain C., Besson R., Legris A. (2002). Atomic-scale Ab-initio study of the Zr-H system: I. Bulk properties. Acta Mater..

[96] Nazarov R., Majevadia J.S., Patel M., Wenman M.R., Balint D.S., Neugebauer J., Sutton A.P. (2016). First-principles calculation of the elastic dipole tensor of a point defect: Application to hydrogen in *α*-zirconium. Phys. Rev. B.

[97] Fukai Y. (2006). The Metal-Hydrogen System: Basic Bulk Properties.

[98] Cordero B., Gómez V., Platero-Prats A.E., Revés M., Echeverría J., Cremades E., Barragán F., Alvarez S. (2008). Covalent radii revisited. Dalton Trans..

[99] Blackmur M.S., Preuss M., Robson J.D., Zanellato O., Cernik R.J., Ribeiro F., Andrieux J. (2016). Strain evolution during hydride precipitation in Zircaloy-4 observed with synchrotron X-ray diffraction. J. Nucl. Mater..

[100] Weekes H., Jones N., Lindley T., Dye D. (2016). Hydride reorientation in Zircaloy-4 examined by in situ synchrotron X-ray diffraction. J. Nucl. Mater..

[101] Wang S., Giuliani F., Britton T.B. (2019). Microstructure and formation mechanisms of *δ*-hydrides in variable grain size Zircaloy-4 studied by electron backscatter diffraction. Acta Mater..

[102] Bair J., Zaeem M.A., Tonks M. (2015). A review on hydride precipitation in zirconium alloys. J. Nucl. Mater..

[103] Heo T.W., Colas K.B., Motta A.T., Chen L.Q. (2019). A phase-field model for hydride formation in polycrystalline metals: Application to *δ*-hydride in zirconium alloys. Acta Mater..

[104] Vizcaíno P., Santisteban J., Alvarez M.V., Banchik A., Almer J. (2014). Effect of crystallite orientation and external stress on hydride precipitation and dissolution in Zr2.5. J. Nucl. Mater..

[105] Tikare V., Weck P.F., Mitchell J.A. (2015). Modeling of Hydride Precipitation and re-Orientation.

[106] Blat M., Noel D. (1996). Detrimental role of hydrogen on the corrosion rate of zirconium alloys. Zirconium in the Nuclear Industry: Eleventh International Symposium.

[107] Shinohara Y., Abe H., Iwai T., Sekimura N., Kido T., Yamamoto H., Taguchi T. (2009). In situ TEM observation of growth process of zirconium hydride in Zircaloy-4 during hydrogen ion implantation. J. Nucl. Sci. Technol..

[108] Krishna K.M., Sain A., Samajdar I., Dey G., Srivastava D., Neogy S., Tewari R., Banerjee S. (2006). Resistance to hydride formation in zirconium: An emerging possibility. Acta Mater..

[109] Škarohlíd J., Ashcheulov P., Škoda R., Taylor A., Čtvrtlík R., Tomáštík J., Fendrych F., Kopeček J., Cháb V., Cichoň S. (2017). Nanocrystalline diamond protects Zr cladding surface against oxygen and hydrogen uptake: Nuclear fuel durability enhancement. Sci. Rep..

[110] Youssef M., Yang M., Yildiz B. (2016). Doping in the Valley of Hydrogen Solubility: A Route to Designing Hydrogen-Resistant Zirconium Alloys. Phys. Rev. Appl..

